# *Arabidopsis thaliana* MIRO1 and MIRO2 GTPases Are Unequally Redundant in Pollen Tube Growth and Fusion of Polar Nuclei during Female Gametogenesis

**DOI:** 10.1371/journal.pone.0018530

**Published:** 2011-04-11

**Authors:** Christopher G. Sørmo, Tore Brembu, Per Winge, Atle M. Bones

**Affiliations:** Department of Biology, Norwegian University of Science and Technology (NTNU), Trondheim, Norway; University of Melbourne, Australia

## Abstract

MIRO GTPases have evolved to regulate mitochondrial trafficking and morphology in eukaryotic organisms. A previous study showed that T-DNA insertion in the Arabidopsis *MIRO1* gene is lethal during embryogenesis and affects pollen tube growth and mitochondrial morphology in pollen, whereas T-DNA insertion in *MIRO2* does not affect plant development visibly. Phylogenetic analysis of MIRO from plants revealed that MIRO 1 and 2 orthologs in dicots cluster in two separate groups due to a gene/genome duplication event, suggesting that functional redundancy may exists between the two *MIRO* genes. To investigate this possibility, we generated *miro1* **^(+/−)^**/*miro2-2* **^(−/−)^** plants. Compared to *miro1* **^(+/−)^** *plants*, the *miro1* **^(+/−)^**/*miro2-2* **^(−/−)^** plants showed increased segregation distortion. *miro1* **^(+/−)^**/*miro2-2* **^(−/−)^** siliques contained less aborted seeds, but more than 3 times the number of undeveloped ovules. In addition, reciprocal crosses showed that co-transmission through the male gametes was nearly absent, whereas co-transmission through the female gametes was severely reduced in *miro1* **^(+/−)^**/*miro2-2* **^(−/−)^** plants. Further investigations revealed that loss of *MIRO2* (*miro2* **^(−/−)^**) function in the *miro1* **^(+/−)^** background enhanced pollen tube growth defects. In developing *miro1* **^(+/−)^**/*miro2* **^(−/−)^** embryo sacs, fusion of polar nuclei was further delayed or impaired compared to *miro1* plants. This phenotype has not been reported previously for *miro1* plants and coincides with studies showing that defects in some mitochondria-targeted genes results in the same phenotype. Our observations show that loss of function in *MIRO2* in a *miro1* **^(+/−)^** background enhances the *miro1* **^(+/−)^** phenotype significantly, even though *miro2* **^(−/−)^** plants alone does not display any phenotypes. Based on these findings, we conclude that MIRO1 and MIRO2 are unequally redundant and that a proportion of the *miro1* **^(+/−)^**/*miro2* **^(−/−)^** plants haploid gametes displays the complete null phenotype of MIRO GTPase function at key developmental stages.

## Introduction

Mitochondria are main cellular source for energy in eukaryotic cells. Additionally, mitochondria are important for calcium homeostasis, oxidative stress processes, production of metabolic intermediates and programmed cell death (PCD). Mitochondria are highly dynamic organelles that are transported on microtubule/actin structures within the cell. Their dynamic behaviour is also reflected in fusion and fission events that change the number and morphology of mitochondria. In plants, research has elucidated how mitochondria move along cytoskeletal tracks and how mitochondrial fission takes place in plant cell. Still, the molecular events behind mitochondrial fusion are largely unknown in plants [1], [2], [3]. Studies of mitochondrial dynamics in cultured tobacco cells showed that movement mainly is dependent on cytoplasmic actin strands, whereas immobilization is dependent on both actin and microtubules [4]. In contrast to plants, the movement of mitochondria in animal cells mainly occurs along microtubules and is facilitated by kinesins. In neurons, transport along axons is necessary for accumulation of mitochondria in regions with high energy demands. The main players involved in linking kinesin to mitochondria are the MIRO GTPases and Milton [5].

Human MIRO GTPases were discovered through a genome search for RHO consensus domains by Fransson and colleagues [6], and were classified as mitochondrial RHO GTPases. They are atypical to conventional Rho GTPases in possessing two G-domains separated by two calcium binding EF-hand motifs. MIRO GTPases are exposed towards the cytosol, and are connected to the outer membrane of mitochondria through a C-terminal transmembrane domain [6], [7]. The two GTPase domains of Miro lack the typical Rho-specific insert region and have an overall sequence divergence from other Rho GTPases. Thus, MIRO GTPases may be considered to constitute a new subfamily of the Ras superfamily of small GTPases [8]. Orthologs of MIRO GTPases have been discovered in yeast (Gem1p) and Drosophila (dMIRO). Common for these orthologs is their importance in mitochondrial trafficking and morphology [9], [10]. In Drosophila, the adaptor protein Milton binds to MIRO and recruits kinesin heavy chain to form a microtubule transport complex in axons [11]. In humans, two Milton-related proteins (GRIF-1 and TRAK1/OIP106) have been shown to interact with hMIRO through the N-terminal GTPase domain and mediate mitochondrial transport by modulating kinesin activity [12], [13].

The Arabidopsis genome encodes three MIRO GTPases that are predicted to have the same domain organization as MIRO GTPases described in other species. Localization experiments showed that MIRO1 (At5g27540) and MIRO2 (At3g63150) localize to mitochondria through a C-terminal trans-membrane domain [14]. *MIRO1* and *MIRO2* are ubiquitously expressed in all plant tissues, whereas *MIRO3* (At3g05310) shows very low expression in comparison [14]. Further observations revealed that developing embryos homozygous for a T-DNA insertion in *MIRO1* arrests during early stages of development [14]. A recent study shows that aberrant mitochondrial morphology and distribution in *miro1* **^(−/−)^** embryonic cells significantly contributes to the observed developmental arrest. Apical cells in arrested two-celled *miro1* **^(−/−)^** embryos contain significantly less mitochondria compared with wild type cells [15]. Mutation in *MIRO1* also influence pollen germination as well as mitochondrial morphology and streaming during pollen tube growth, which in turn resulted in reduced male genetic transmission of the mutant allele [14]. In the same study two mutant lines with T-DNA insertions in the *MIRO2* gene were studied. Homozygous *miro2* plants showed no apparent mutant phenotypes, suggesting that MIRO2 plays no important role during plant development and that MIRO2 apparently is not functionally redundant to MIRO1.

An Arabidopsis Calcium Binding GTPase (AtCBG) discovered in a screen for EF hands and GTPase domain reported by Jayasekaran and colleagues [16] is actually MIRO2. According to the study, MIRO2 shows calcium dependent GTPase activity and two MIRO2 T-DNA mutants investigated were reported to be sensitive to both NaCl and ABA stress.

Here we show, by generating a *miro1* **^(+/−)^**/*miro2-2* **^(−/−)^** plant, that MIRO2 is unequally redundant to MIRO1 during specific stages of gametophyte development and function. Unequal genetic redundancy is defined as a phenomenon where loss of function in one gene produces mutant phenotypes, whereas a mutant with loss of function in the paralogous gene does not display any phenotypes. Importantly, loss of function in both paralogous genes results in strong enhancement of the initial phenotype [17]. Our results show that crossing of *miro1* **^(+/−)^** and *miro2* **^(−/−)^** produces mutant plants with enhanced *miro1* **^(+/−)^** phenotypes and that a proportion of the developing haploid male and female gametes display the full null phenotype of MIRO GTPase function.

## Methods

### Gene expression and phylogenetic analysis

For gene expression analysis, transcriptome data were obtained from the Arabidopsis eFP browser [18] and visualized using Microsoft Excel 2003.

Plant MIRO sequences were downloaded from the NCBI database. Due to lack of annotation and wrong gene models, 15 of the MIRO proteins were deduced from genomic and overlapping ESTs. Predicted protein sequences were imported into the ClustalX program [19] and a pair wise alignment was made using the Gonnet 250 score matrix. The resulting protein alignment was exported as a MSF file and imported into the GeneDoc program [20] for manual editing. The edited alignment was re-imported into ClustalX and a bootstrapped neighbour joining (NJ) tree was made running 1000 bootstrap trials. A rooted phylogenetic tree was constructed with the TreeView program [21], where the *Physcomitrella patens* PpMIRO2 was used as an outgroup. Accession numbers for the various Miro GTPases are listed in File S1.

### Plant growth conditions

Seeds were surface-sterilized using vapor phase chlorine gas for 3–4 hours and plated onto half strength Murashige-Skoog medium, pH 5.8, 0.6% (w/v) agar. The growth media was supplemented with 25 µg/ml Kanamycin (*miro2-2*) and/or 10 µg/ml BASTA (*miro1*). Seeds were vernalized for 48 hours before germination at 22°C, 16-h light and 18°C, 8-h dark conditions. 7 DAG selection resistant seedlings were transferred to soil and grown under the same conditions as above.

### *miro* T-DNA mutants; identification and crosses

The *miro2-2* (SALK_157090) plants were backcrossed into Col-WT background before it was crossed with *miro1* (*emb2473*) plants; thus *miro2-2* was backcrossed twice and *miro1* once. Genomic DNA was isolated using SP Plant Mini Kit (Omega) and REDExtract-N-AMP Plant PCR Kit (Sigma) was used for the segregation analysis.

**Figure 1:**
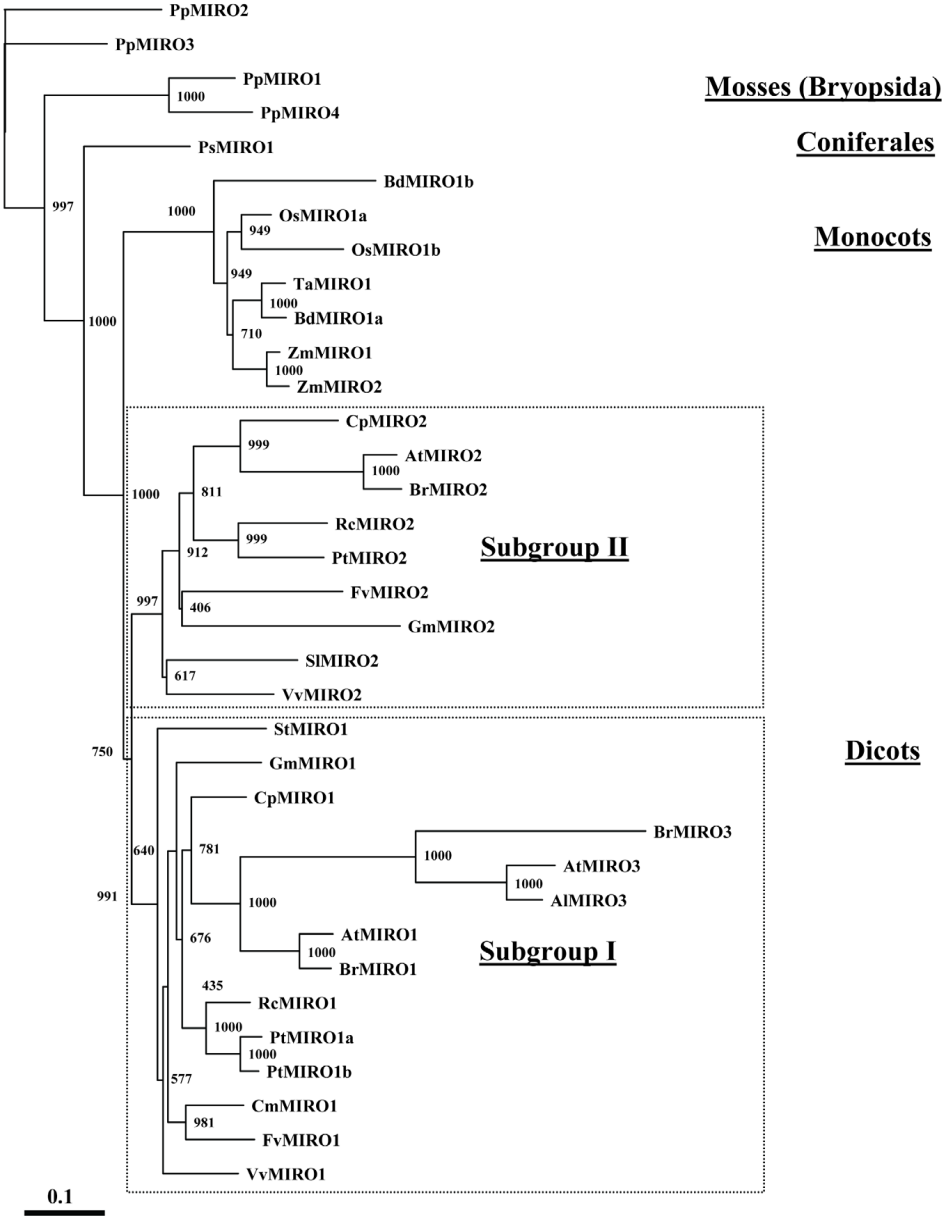
Phylogenetic tree of MIRO GTPases in Embryophyta. Phylogenetic tree based on protein sequence alignment of MIRO GTPases from plants. The tree is rooted with a *Physcomitrella patens* MIRO ortholog as an outgroup. Numbers indicate bootstrap values. Dashed line boxes enclose the two MIRO ortholog subgroups in dicots. Abbreviations: At- *Arabidopsis thaliana*, Al- *Arabidopsis lyrata*, Bd- *Brachypodium distachyon*, Br- *Brassica rapa*, Cm- *Cucumis melo*, Cp- *Carica papaya*, Fv- *Fragaria vesca*, Gm- *Glycine max*, Os- *Oryza sativa* (Japonica), Pp- *Physcomitrella patens*, Ps- *Picea sitchensis*, Pt- *Populus trichocarpa*, Rc- *Ricinus communis*, Sl- *Solanum lycopersicum*, St- *Solanum tuberosum*, Ta- *Triticum aestivum*, Vv- *Vitis vinifera*, Zm- *Zea mays*.

The various mutant T-DNA insertions were verified using PCR with T-DNA specific primers and gene specific primers (Figure 1B and C); *miro1*: (WT) 5′-CAGGAATCAACTACTGATGAGC3′ and 5′-CCAGTTGCTTGTAGAAGTTGCA-3′, (T-DNA) 5′-CCAGTTGCTTGTAGAAGTTGCA-3′ and 5′-GCATCTGAATTTCATAACCAATC-3′; *miro2-2*:(WT) 5′-GTTAGTAGCAAAAGTCTGAACT-3′ and 5′-GGGTTCTCTGCTGTACTCACGA-3′, (T-DNA) 5′-GTTAGTAGCAAAAGTCTGAACT-3′ and 5′-CGGAACCACCATCAAACAGGAT-3′.

### Phenotypical analysis

Mature siliques from the same positions along the main inflorescence were measured for length and dissected to identify aborted ovules and embryo lethality. The 5 first siliques on the main inflorescence were avoided for this analysis. Pollen viability test using Alexander stain was performed as described in [22]. Mature pollen nuclei were stained using 1 µg/ml DAPI in extraction buffer (0.1% Nonidet P40, 10% DMSO, 50 mM PIPES pH 6.9, 5 mM EGTA pH 7.5). Pollen germination assays were performed as described in [22] and germinated over night. Germinated pollen was stained over night at 37°C with 1 mg/ml X-Gluc solution containing 50 mM Na_3_PO_4_, 0.5 mM K_3_Fe(CN)_6_, 0.5 mM K_4_Fe(CN)_6_, 10 mM EDTA, 0.01% Triton X-100 and 10% (w/v) sucrose. For embryo sac analysis, siliques were cleared over night in Hoyer's solution. Images were acquired with a Nikon E800 microscope/Nikon DsRi1 camera using NisElements F software. Pollen tube lengths were measured using ImageJ [23] software. Images were processed using Adobe Photoshop Elements 4.0.

## Results

### Evolution of MIRO GTPases within Embryophyta

Database searches indicates that MIRO GTPases exist in Metazoa, Fungi, Rhodophyta, Stramenopiles, Alvoelata, Heterolobosea, Euglenozoa, Mycetozoa and Viridiplantae, whereas they are missing from the anaerobic Entamoebidae and Parabasalia that lack mitochondria all together, suggesting that MIRO GTPases are only found organisms that contain mitochondria. However, MIRO GTPases are not present in Haptophyceae that contain mitochondria, which indicate that MIRO GTPases are not required in some forms of eukaryotic life [24]. A phylogenetic analysis of MIRO proteins in Embryophyta was performed based on protein primary structure alignments, and the phylogentic relationship between 35 MIRO proteins was visualized as a phylogram rooted with a *Physcomitrella patens* MIRO ortholog as an outgroup (Figure 1). In Embryophyta, MIRO GTPases are found in mosses, Coniferales, monocots and dicots. In dicots, the paralog MIRO genes (MIRO1 and MIRO2) cluster into two distinct MIRO subgroups (I & II) with bootstrap confidence levels above 99%.

The origin of the MIRO paralogs in dicots is due to a gene/genome duplication event that occurred after the diversification of monocots and eudicots. Additionally, sometime during evolution of the Brassicaceae family an additional duplication event within MIRO subgroup I resulted in development of the MIRO3 paralogs that show a rapid divergent evolution compared to other subgroups.

Since paralogous genes often have the same or similar function, it is likely that MIRO paralogs may display some degree of functional redundancy during plant development.

Yamaoka and Leaver report that the two paralogs *MIRO1* and *MIRO2* are expressed in all plant tissues investigated, implying functional roles during plant growth and all developmental stages. However, neither *miro1* **^(+/−)^** nor *miro2* **^(−/−)^** T-DNA mutants shows developmental defects during sporophytic growth [14].

**Figure 2:**
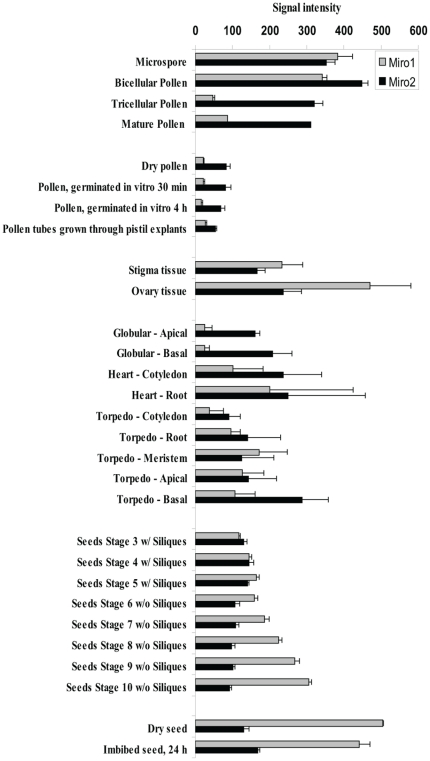
Gene expression of Arabidopsis MIRO1 and MIRO2 in different plant tissues. Note the difference in expression levels between MIRO1 and MIRO2 during pollen development, especially in mature pollen and during pollen germination. During embryo development there are also both overlapping and quantitative differences in between *MIRO1* and *MIRO2* gene expression. Data used were retrieved from the Arabidopsis eFP browser [18]. Values are means, +SD.

To investigate quantitative expression differences between *MIRO1* and *MIRO2* during gametophyte development closer, we used the Arabidopsis eFP browser [18]. The *in silico* analysis revealed that both *MIRO1* and *MIRO2* are expressed in most gametophyte tissues and stages (Figure 2). Comparing these expression profiles with the *miro1* **^(+/−)^** phenotypes reported by Yamaoka and Leaver [14], it is striking that *MIRO2* shows higher expression at the globular stage and the following stages during embryo development compared to *MIRO1*. The *miro1* **^(−/−)^** embryos abort early during embryo development, between the zygote and the four-terminal-cell stage. However, data from the Arabidopsis eFP browser does not contain any expression data from these stages. Still, these findings indicate that MIRO2 may be functionally redundant to MIRO1 during embryo development. Yamaoka and Leaver [14] also reports that *miro1* pollen show reduced germination rate and pollen tube growth compared to wild type pollen. The expression data presented here shows that during pollen development and germination, *MIRO2* has higher expression levels compared to *MIRO1* and clearly suggests that MIRO2 could be functionally redundant to MIRO1.

Interestingly, *MIRO3* shows very high expression in both chalazal and peripheral endosperm during seed development (from pre-globular to heart stage) with up to 110 and 80 fold higher expression levels compared to *MIRO1* and *MIRO2*, respectively (Data from Arabidopsis Seed eFP browser) [25]. This expression pattern suggests that within Brassicaceae, MIRO3 orthologs may have evolved to function mainly in endosperm development.

Considering the evolution of eudicot MIRO GTPases, the expression pattern divergence during gametophyte development and the absence of phenotype in the *miro2* **^(−/−)^** T-DNA plants, we wanted to investigate if unequal genetic redundancy exits between the MIRO1 and MIRO2 paralogs in Arabidopsis. By generating *miro1* **^(+/−)^**/*miro2* **^(−/−)^** plants it should be possible to discern if genetic redundancy between the MIRO1 and MIRO2 paralogs exists. Importantly, if genetic redundancy exists this should be manifested as novel or enhanced *miro1* **^(+/−)^** phenotypes.

### *miro* T-DNA mutants

**Figure 3:**
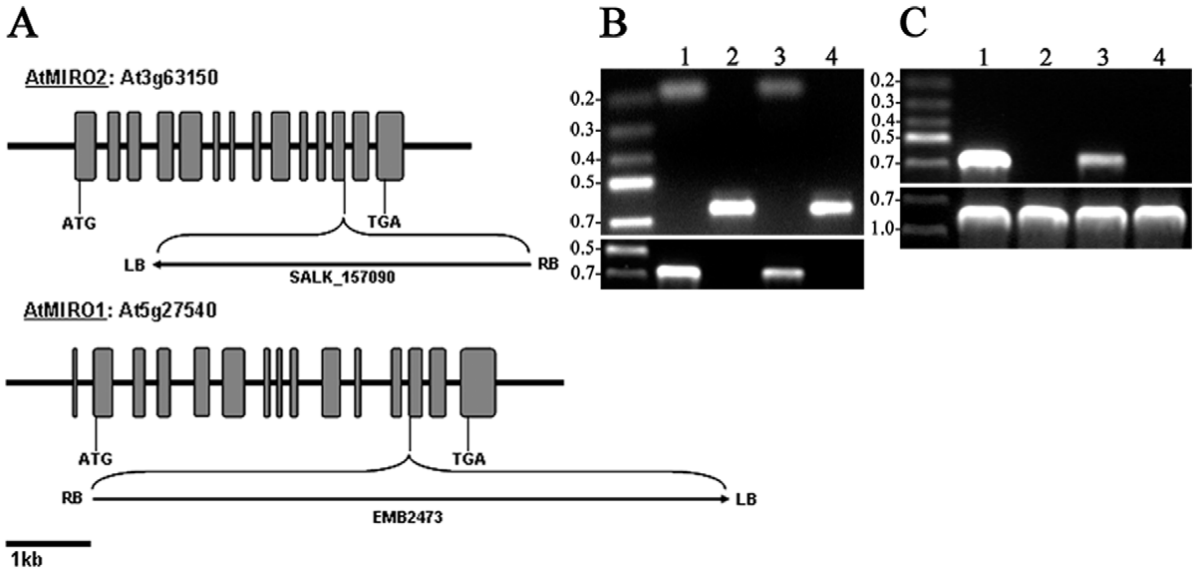
Characterization of MIRO T-DNA mutants. (**A**) A schematic overview of the *MIRO1* and *MIRO2* gene structures and the position and orientation of the T-DNA insertion sites within the genes. Closed gray boxes indicate exons. (**B**) Genotyping of *MIRO* T-DNA mutants. **1**: *miro1* **^(+/−)^**, **2**: *miro2-2* **^(−/−)^**, **3**: *miro1* **^(+/−)^**/*miro2-2* **^(−/−)^**, **4**: *miro1* **^(+/−)^**/*miro2-2* **^(−/−)^**. Top panel: Verification of T-DNA insertions using gene and T-DNA specific primers Bottom panel: Verification of WT allele. Underline: allele investigated. (**C**) Genotyping primer control using Col-WT gDNA. Top panel: **1**: *MIRO1* WT allele primers, **2**: *miro1* T-DNA primers, **3**: *MIRO2* WT allele primers, **4**: *miro2-2* T-DNA primers. Bottom panel: 18s ribosomal RNA PCR control. **1**: *miro1* ^(+/−)^, **2**: *miro2-2* ^(−/−)^, **3**: *miro1* **^(+/−)^**/*miro2-2* **^(−/−)^**, **4**:Col.

In order to study the functional relationship between MIRO GTPases in Arabidopsis, we obtained independent mutant lines from publicly available seed collections. *miro1*/*emb2473* was obtained from the Seed Genes Project [26] and *miro2*/SALK_157090 was obtained from the SALK collection [27]. These two mutant lines are the same as those studied by Yamaoka and Leaver. Both lines are in the Columbia background (Col-0) and are henceforth designated as *miro1* and *miro2-2* respectively [14]. The *miro1* and *miro2-2* plants harbour T-DNA insertions in the beginning and the end of the 12th exon of *MIRO1* and *MIRO2*, respectively (Figure 3A). To investigate whether genetic redundancy between the *MIRO1* and *MIRO2* genes exits, we crossed a heterozygous *miro1* **^(+/−)^** plant with a *miro2-2* **^(−/−)^** plant in order to possibly obtain *miro1* **^(+/−)^**/*miro2-2* **^(−/−)^** plants.

Segregation analysis of self-pollinated *miro1* ^(+/−)^ plants showed that 57.1% (Table 1) of the progeny were viable on MS media supplemented with BASTA, which concurs with Yamaoka and Leavers observations [14]. For self-pollinated *miro1* **^(+/−)^**/*miro2-2* **^(+/−)^** plants from the crossings we expected 37.5% (10∶6) viable progeny on MS media supplemented with BASTA (*miro1*) and kanamycin (*miro2-2*). Since *MIRO1* and *MIRO2* are located on two separate chromosomes, one would expect that if the T-DNA insertions in the *MIRO2* locus do not contribute to gametophyte development and function, they would segregate independently from the *miro1* allele.

**Table 1:** Segregation analysis of the *miro1* and *miro2-2* alleles.

Parental genotype	Seed germ. (%)	Total seeds	Selection^R^	Selection^S^	Selection^R^ (%)	Hypothesis	χ^2^	*P*(*P*<0.05)
*miro1* ^(+/−)^/*miro2-2* ^(+/−)^×*miro1* ^(+/−)^/*miro2-2* ^(+/−)^	93.2	502	140	328	29.9	3∶5	11.489	0.0007
*miro1* ^(+/−)^/*miro2-2* ^(−/−)^×*miro1* ^(+/−)^/*miro2-2* ^(−/−)^	94,4	1026	163	805	16.8			
*miro1* ^(+/−)^×*miro1* ^(+/−)^	89.9	614	315	237	57.1			
*miro2* ^(−/−)^×*miro2* ^(−/−)^	97.1	593						
Colombia WT	97.1	414						

Selection^R^ (Seedlings with resistance to selection agent): *miro1* ^(+/−)^/*miro2-2* ^(−/−)^; BASTA/Kanamycin, miro1/MIRO1; BASTA, *miro2-2*; Kanamycin. Selection^S^: Seedlings with sensitivity to selection agent.

If so, expected segregation of *miro1* **^(+/−)^**/*miro2-2* **^(−/−)^** alleles from self-pollinated *miro1* **^(+/−)^**/*miro2-2* **^(+/−)^** plants would be 33.3% (2∶1) within all progeny resistant to selection agents. Notably, no *miro1* **^(−/−)^**/*miro2-2* **^(−/−)^** progeny will be formed during self-fertilization of *miro1* **^(+/−)^**/*miro2-2* **^(+/−)^** plants. However, segregation analysis (Table 1) showed that 29.9% of the progeny from self-pollinated *miro1* **^(+/−)^**/*miro2-2* **^(+/−)^** plants were resistant to both selection agents. This is significantly lower than the expected 37.5% (*P* value = 0.0007) and suggested that additional loss of function in MIRO2 has an additional effect on gametophyte development or function. To validate this finding further, we genotyped the progeny from the self-fertilized *miro1* **^(+/−)^**/*miro2-2* **^(+/−)^** plants. PCR analysis (File S2) of 80 individual plants grown on selective media showed that 17 plants (21.3%) were *miro1* **^(+/−)^**/*miro2-2* **^(−/−)^** plants. This result deviates significantly from the 2∶1 hypothesis (*P* value = 0.0218) and clearly indicates that the two alleles do not segregate independently.

From self-pollinated *miro1* **^(+/−)^**/*miro2-2* **^(−/−)^** plants however, only 16.8% of the germinating progeny were resistant to both selection agents and viable on MS media. In comparison, 57.1% of the *miro1* **^(+/−)^** plants were resistant to BASTA. Taken together, the segregation analysis of the *miro1* and *miro2-2* alleles clearly indicates that a T-DNA insertion in the *MIRO2* locus does not segregate independently of the *miro1* locus, but rather that there is some level of functional redundancy between the *MIRO1* and *MIRO2* genes.

### The *miro1* ^(+/−)^/*miro2-2* ^(−/−)^ plants show increased gametophytic defects

**Figure 4:**
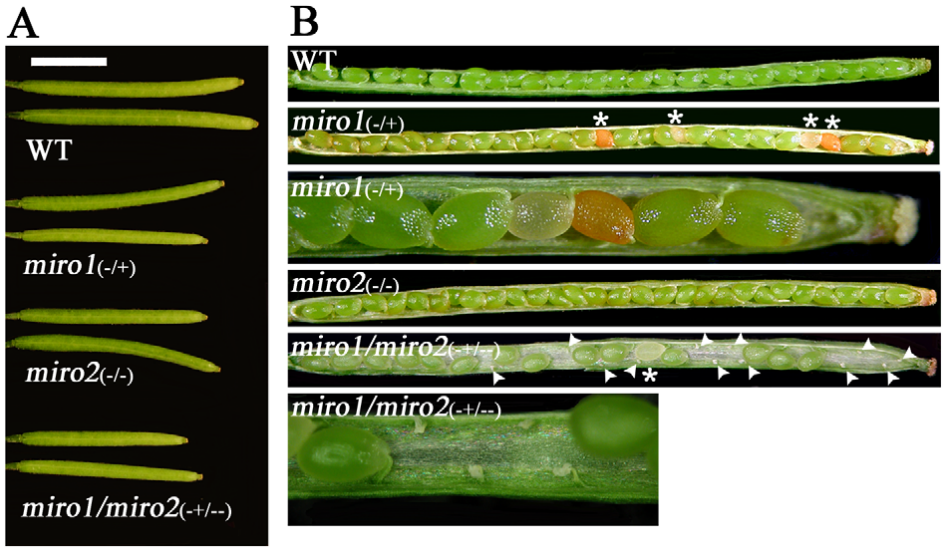
Silique size and embryo development in *miro* plants. A: Siliques from wild type and *miro* plants grown simultaneously and under equal conditions. Siliques are from the same positions along the main inflorescence. Scale bar: 0.5 cm. B: Open *miro1* **^(+/−)^**/*miro2-2* **^(−/−)^** siliques contain a larger number of undeveloped ovules and fewer terminated embryos compared to the *miro1* **^(+/−)^** siliques. Asterisks indicate terminated embryos and arrowheads indicate undeveloped ovules. Picture 3 and 6 from the top are higher magnification of the siliques from *miro1* **^(+/−)^** and *miro1* **^(+/−)^**/*miro2-2* **^(−/−)^**,respectively.

During sporophyte development, no visible phenotypes were observed in *miro1*/*miro2-2* heterozygous plants or the *miro1* **^(+/−)^**/*miro2-2* **^(−/−)^** plants. A closer investigation of siliques from the *miro1* **^(+/−)^**/*miro2-2* **^(−/−)^** plants showed that the siliques are significantly shorter compared to both wild type plants and the individual *miro* plants (Figure 4A). The length of siliques collected from the same positions of the main inflorescence of wild type and *miro* plants was measured and an unpaired Student's T-tests analysis was performed. T-tests showed significant differences (P<0.0001) in silique length between WT-Col (1.33 cm, SD = 0.056 cm, n = 10), *miro1* **^(+/−)^**/*miro2-2* **^(−/−)^** (1.11 cm, SD = 0.04 cm, n = 10) and *miro1* (1.22 cm, SD = 0.038 cm, n = 10) (results shown are representative data from one of three separate experiments and each experiment showed significant differences in comparison of silique length). We believe that this phenotype is not of sporophytic origin but that it may be a result of a lower degree of fertilization in mutant plants.

Yamaoka and Leaver reported 10% unfertilized ovules and 13% aborted seeds within *miro1* siliques [14]. During our experiments we observed similar numbers, with 7.4% unfertilized ovules and 17.2% aborted seeds (n = 1318) in *miro1* siliques (Table 2). In contrast, the *miro1* **^(+/−)^**/*miro2-2* **^(−/−)^** plants (Figure 4B) produced siliques with 34.5% unfertilized ovules and 3.4% aborted seeds (n = 1165) randomly dispersed inside the silique, which indicate that the *miro1* **^(+/−)^**/*miro2-2* **^(−/−)^** plant has an increased impact on male and/or female gametogenesis and/or gamete function compared to *miro1* **^(+/−)^** plants.

**Table 2:** Silique analysis of *miro* plants.

	Wild Type	*Miro2-2* ^(−/−)^	*miro1* ^(+/−)^	*miro1* ^(+/−)^/*miro2-2* ^(−/−)^
**Total # of embryos**	642	1135	1318	1165
**Unfertilized ovules (n)**	**1.7%** (11)	**1.4%** (16)	**7.4%** (98)	**34.5%** (402)
**Embryo lethal (n)**	**0.8%** (5)	**0.5%** (6)	**17.2%** (226)	**3.4%** (40)
**Total lethality**	**2.5%**	**2%**	**24.6%**	**37.9%**
**Seed set/silique (n)**	**57** (11)	**53** (21)	**43.2** (23)	**36.1** (20)

Furthermore, *miro1* **^(+/−)^**/*miro2-2* **^(−/−)^** siliques contained less aborted seeds than *miro1* **^(+/−)^** siliques. The background of this phenotype was further studied by co-transmission efficiency (TE) analysis of the mutant alleles. Reciprocal crosses showed that co-transmission (TE: selection^R^/selection^S^) of both *miro* alleles through the male gametes was 0.12% (n = 796); through the female gametes the co-transmission efficiency was 34.7% (n = 625, % of total seedlings: 25.8%). These co-transmission efficiencies are significantly lower than what was reported for the transmission *miro1* allele alone (12.8% and 75.2%, respectively) [14].

The severe impact of *miro2-2* allele on male genetic transmission in the *miro1* background means that formation of homozygous *miro1* embryos rarely occurs in the *miro1* **^(+/−)^**/*miro2-2* **^(−/−)^** siliques, thereby explaining the reduction of aborted seeds in the *miro1* **^(+/−)^**/*miro2-2* **^(−/−)^** plants. This also implies that most of the observed undeveloped ovules may be a result of impaired female gametophyte development caused by maternally inherited *miro1/miro2-2* alleles. However, the penetrance of the female gametophyte defect is not complete since 16.8% of the offspring carry both *miro1/miro2-2* alleles. Incomplete penetrance is not an uncommon phenomenon and has been reported for other mutants affected in female gametophyte development as well [28].

### Loss of function in MIRO2 enhances pollen tube growth defects in the *miro1* ^(+/−)^ background

The low co-transmission efficiency through the male gamete suggests aberrant pollen development, germination and/or tube growth. Previous studies showed that pollen from *miro1* **^(+/−)^** plants matured normally, but that both pollen germination and tube growth was impaired [14].In *miro1* **^(+/−)^**/*miro2-2* **^(−/−)^** plants, half of the developing male gametes carry the *miro1 and miro2* T-DNA alleles, which could possibly lead to defects in pollen development. This notion is supported by the fact that *MIRO2* shows higher expression levels compared to *MIRO1* during male gametophyte development and tube growth.

**Figure 5:**
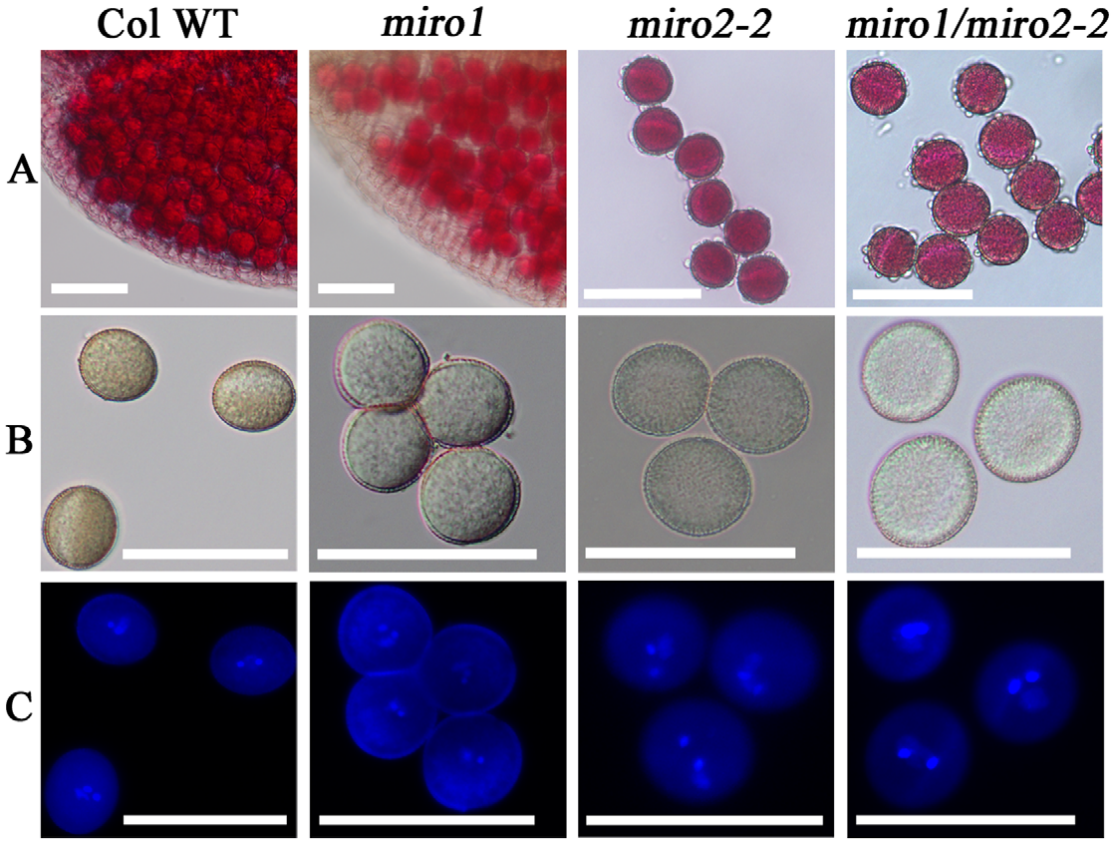
Pollen viability and development. A: Viability test using Alexander's stain. For Col-WT and *miro1*, anthers were fixed and stained. B: DIC images. Note that the *miro1* plants are in the *quartet* background (*quartet1* **^(−/−)^**: At5g55590), which is outcrossed in the *miro1*/*miro2-2* pollen. C: DAPI staining (same as B) shows that mature *miro1/miro2-2* pollen are correctly differentiated with two brightly stained sperm nuclei and one diffusely stained vegetative nucleus. Scale bar: 50 µm.

A pollen viability test using Alexander's stain was performed and showed that all of the mature pollen from *miro1* **^(+/−)^**/*miro2-2* **^(−/−)^** plants were viable (Figure 5A). Mutant pollen was morphologically undistinguishable from wild type pollen (Figure 5B). Nuclear staining with DAPI showed that the pollen developed normally and reached maturity with two sperm cell nuclei and a vegetative nucleus (Figure 5C). We therefore conclude that homozygous loss of MIRO2 function in *miro1* **^(+/−)^** background does not give an additional effect on pollen development and viability.

**Figure 6:**
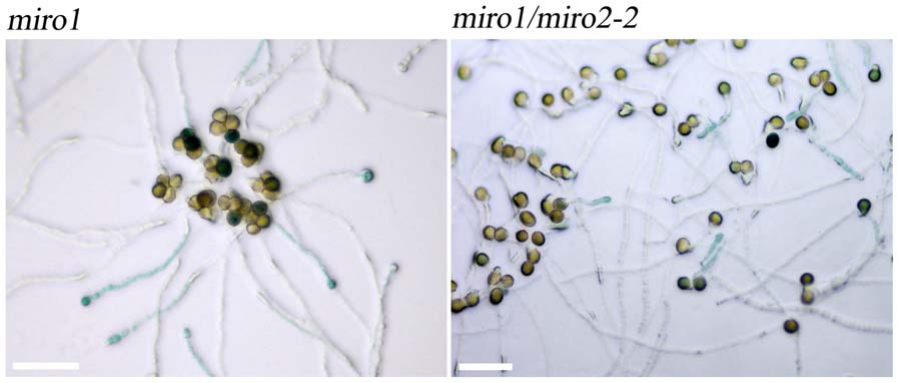
Additional loss of function in MIRO2 enhances pollen tube growth defects in the *miro1* background. Pollen germinated on solid medium for 17 hours and stained with X-Gluc. Scale bar: 100 µm.

The pCSA110 T-DNA insertion in *miro1* **^(+/−)^** plants contains the *GUS* reporter gene regulated by the pollen-specific LAT52 promoter, making distinction between mutant and wild type pollen possible [29]. Pollen from *miro1* **^(+/−)^** and *miro1* **^(+/−)^**/*miro2-2* **^(−/−)^** flowers were collected and germinated on solid pollen media and stained with X-Gluc solution to assess if loss of MIRO2 function in the *miro1* **^(+/−)^** background affects pollen tube growth. GUS negative pollen from both *miro1* **^(+/−)^** and *miro1* **^(+/−)^**/*miro2-2* **^(−/−)^** appeared to grow normally. As expected from previous results, GUS positive pollen in *miro1* **^(+/−)^** showed reduced germination and tube growth [14]. The *miro1* **^(+/−)^**/*miro2-2* **^(−/−)^** plants showed highly significant (P<0.0001) additional impairment of pollen tube growth compared to the *miro1* **^(+/−)^** alone (Figure 6). GUS positive pollen tubes from *miro1* **^(+/−)^** grew to an average of 436.2 µm (SD = 136.0 µm, n = 133) whereas GUS positive pollen tubes from *miro1* **^(+/−)^**/*miro2-2* **^(−/−)^** plants grew to an average of 178.3 µm (SD = 84.8 µm, n = 209) after 17 hours of growth (results shown are data from 4 separate experiments). All in all, these observations clearly indicate that loss of MIRO2 function in a *miro1* **^(+/−)^** background does not affect pollen development but has an additional strong negative effect on pollen tube growth.

### *miro1* ^(+/−)^/*miro2-2* ^(−/−)^ plants are affected in embryo sac development

**Figure 7:**
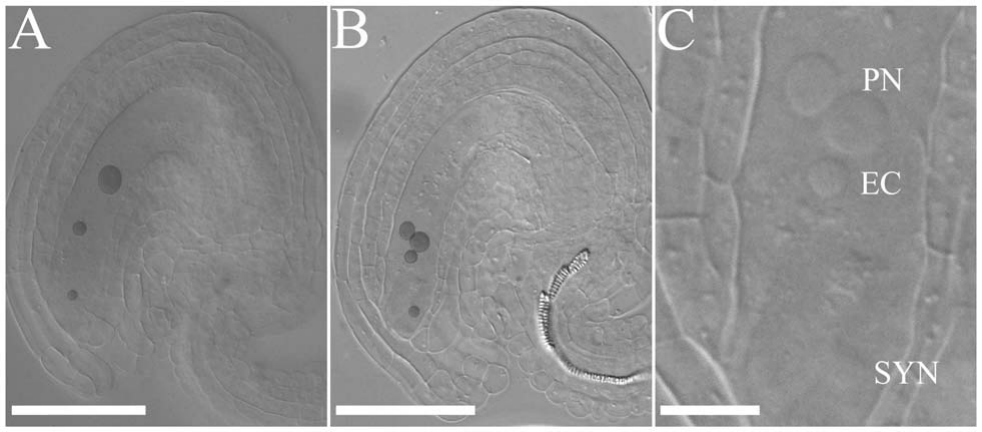
*miro1*/*miro2-2* female gametophytes are affected during fusion of polar nuclei. Phenotypes of *miro1* **^(+/−)^**/*miro2-2* **^(−/−)^** female gametophytes 48 hours after emasculation. A: Normal mature embryo sac. B: The polar nuclei have failed to fuse. C: Higher magnification of B (PN; Polar nuclei, EC; Egg cell, SYN; Synergid). Contrast of nuclei (except in C) has been artificially enhanced. Scale bar: 50 µm except in C: 10 µm.

Since co-transmission of both *miro* alleles through the male gametophyte is nearly absent, the observed undeveloped ovules must be due to a combined effect of the *miro1/miro2-2* alleles during female gametophyte development. To investigate closer at what stage the undeveloped ovules are affected, both *miro1* **^(+/−)^**/*miro2-2* **^(−/−)^** and *miro1* **^(+/−)^** flowers were emasculated, and the siliques were cleared and observed with DIC-microscopy after 48 hours. In ovules from the *miro1* **^(+/−)^** plant, 19.0% (n = 327) of the embryo sacs displayed two slightly larger nuclei localized adjacent to each other in addition to both egg cell nuclei and synergid cell nuclei. This phenotype was interpreted as a defect or delay during fusion of the polar nuclei (karyogamy) (Figure 7B). In *miro1* **^(+/−)^**/*miro2-2* **^(−/−)^** plants we observed that 43.1% (n = 418) of the ovules displayed embryo sacs with defects in fusion of polar nuclei. The remainder of the ovules from *miro1* **^(+/−)^** and *miro1* **^(+/−)^**/*miro2-2* **^(−/−)^** plants and all ovules from WT plants (n = 228), had an embryo sac with a normal cellular constitution (one enlarged central cell nucleus, one egg cell nucleus and synergid cell nuclei) (Figure 7A). This defect or delay in fusion of polar nuclei indicates that both MIRO1 and MIRO2 play a role during karyogamy. Karyogamy occurs three times during the lifecycle of angiosperms: once during embryo sac development when the two polar nuclei fuse to form the central cell nucleus and twice during fertilization, where the two sperm cell nuclei fuse with the egg cell and central cell nuclei [28].

Crosses of *miro1* **^(+/−)^**/*miro2-2* **^(−/−)^** plants (female) with wild type pollen showed a co-transmission efficiency of 34.7%, approximately twice of what is observed with self-fertilized *miro1* **^(+/−)^**/*miro2-2* **^(−/−)^** plants. This result strongly indicates that pollen carrying wild type MIRO1 and MIRO2 are able to fertilize and thereby “salvage” some mutant ovules during fertilization. In this case, where male co-transmission is close to zero, it is therefore reasonable to assume that some of the structures that are observed as undeveloped ovules in *miro1* **^(+/−)^**/*miro2-2* **^(−/−)^** siliques are fertilized ovules that are arrested during or shortly after fertilization. In self-fertilized *miro1* **^(+/−)^**/*miro2-2* **^(−/−)^** plants, ovules are mainly fertilized by MIRO1/*miro2-2* pollen. Homozygous *miro1* embryos rarely forms and the defects in fertilization/early embryo development may be an additional effect of the paternally inherited *miro2-2* allele. This is also in line with the increase in aborted embryos in *miro1* **^(+/−)^**/*miro2-2* **^(−/−)^** siliques (3.4% versus 0.8% in Col-wild type).

## Discussion

Our results show that MIRO1 and MIRO2 are unequally redundant in function and that both genes affect pollen tube growth, fusion of polar nuclei during embryo sac development and possibly also nuclei fusion during fertilization. A total loss of MIRO2 function in heterozygous *miro1* **^(+/−)^** background results in enhanced *miro1* phenotypes. Even though MIRO2 initially appeared to be dispensable in gametophyte function, ovule development and embryo development compared to MIRO1, it has retained a significant functional role. In an evolutionary context, this fact may be the reason for maintaining a genomic copy of *MIRO2*, which is manifested as unequal genetic redundancy.

Unequal genetic redundancy is in part attributed to differences in expression patterns and/or expression levels between paralogous genes [17], [30]. In the case of *MIRO1* and *MIRO2*, expression levels are both overlapping and quantitatively different in key developmental stages where phenotypes are present in both *miro1* **^(+/−)^** and *miro1* **^(+/−)^**/*miro2-2* **^(−/−)^** plants (Figure 2).

Contradictory to the observed lack of phenotype in *miro2* ***^(−/−)^*** plants, *MIRO2* shows higher expression compared to *MIRO1* in male gametophytic tissues and several of the embryonic stages (Figure 2). One would expect that loss of function in MIRO2 alone would result in deleterious phenotypes at these developmental stages.

The fact that *MIRO1* and *MIRO2* shows quantitative divergence in expression is indicative of the following fates of the paralogous genes after the duplication; A) neofunctionalization, where the duplicated genes gain a novel function, or B) subfunctionalization, where the function is sub-divided between the two paralogs. Notably, in the latter scenario, both of the paralogous genes represent the total function of the two genes [30], [31]. After duplication, both the regulatory and coding sequences of the paralogous genes may acquire mutations or be subjected to epigenetic effects that affect both the functions and expression patterns of the genes. In support of this assumption, statistical analysis of the expression pattern of 280 phylogenetically identified paralogous pairs in Arabidopsis revealed that 85% of the pairs showed differential expression levels depending on the organ investigated. These findings suggest that mutations in *cis*-acting elements in the promoter regions of the gene pairs contribute to the observed expression pattern shifts. Therefore it is believed that regulatory subfunctionalization and/or neofunctionalization will in part be responsible for the maintenance of the paralogous pair over time [30].

The expression pattern shifts between *MIRO1* and *MIRO2* (Figure 2) supports a hypothesis where a genomic copy of *MIRO2* is retained since it may have undergone regulatory subfunctionalization and/or neofunctionalization after duplication. However, one can not rule out the possibility that MIRO2 also have accumulated mutations in coding regions, resulting in functional subfunctionalization and/or neofunctionalization. Thus, MIRO2 may not have the same level of protein activity as MIRO1, which could explain why *miro2* ***^(−/−)^*** plants do not display any phenotype. In a *miro1* **^(+/−)^**/*miro2-2* **^(−/−)^** setting, however, the cumulative protein activity of the gene pair is below a certain threshold that results in enhanced *miro1* phenotypes [17].

Finally, it should be noted that plants grown under optimal condition in the laboratory does not reflect the various environmental conditions that the plants have been subjected to throughout its evolutionary history. Under certain natural conditions these expression shifts may provide a fitness advantage and therefore result in maintenance of the paralogous pair [32]. This may also be the case for Arabidopsis MIRO2 since it has been implicated in ABA and salt stress [16], which could indicate that MIRO2 have other functional roles compared to MIRO1 during certain environmental conditions. If this is the case, it could explain the difference in the phenotypes between *miro1* ***^(+/−)^*** and *miro2* ***^(−/−)^*** plants during regular growth.

The *miro1/miro2-2* alleles showed very low co-transmission through the male gametes, suggesting aberrant pollen development and/or function. However, our microscopic studies show that there is no additive or new aberrant effect of loss of function of MIRO2 in the *miro1* **^(+/−)^** background, suggesting that loss of function in MIRO1 and MIRO2 does not affect pollen development. This observation is intriguing when taking into consideration that MIRO1 affects mitochondrial morphology in pollen, possibly leading to changes in the intracellular distribution of mitochondria [14]. Furthermore, the fact that metabolic rates in developing pollen are higher compared to sporophyte tissue [33] should warrant the necessity for proper intracellular distribution and morphology of mitochondria during pollen development. Alternatively, male gametophyte development may not be affected due to initial transcription of wild type *MIRO1* in the diploid parental microsporocytes, resulting in sufficient amounts functional protein to rescue developing mutant male gametes in *miro1* **^(+/−)^**/*miro2-2* **^(−/−)^** plants. Such a hypothesis has been put forth by Berg and colleagues [34], in connection with loss of function in aminoacyl-tRNA synthetases predicted to function in mitochondria. As a consequence, mitochondria with wild type MIRO1 are inherited in the daughter cells during meiotic division and therefore proper mitochondrial distribution is sustained during development. However, an additional loss of MIRO2 function in a *miro1* **^(+/−)^** background enhanced pollen tube growth defects compared to single *miro1* **^(+/−)^** plants. All GUS positive pollen tubes from the *miro1* **^(+/−)^**/*miro2-2* **^(−/−)^** plants had significantly reduced tube growth compared to GUS positive *miro1* **^(−)^** pollen tubes (Figure 6). Our hypothesis is that these *miro1* **^(−)^**/*miro2-2* **^(−)^** male gametophytes are not capable of fertilizing ovules due to impaired tube growth, which is confirmed by the reciprocal crosses where co-transmission through the male gametes was nearly absent.

Our data indicate that loss of function in both MIRO1 and MIRO2 affects female gametophyte development during fusion of the polar nuclei. Notably, this phenotype has not been reported earlier for loss of function in plant MIRO GTPases. A fraction of the mutant ovules mature normally, become fertilized and produce viable offspring. Similar to developing pollen, this observation may in part be explained by inheritance of wild type mitochondria with functional MIRO1 from the diploid megasporocyte. Furthermore, the surrounding sporophytic cells could provide sufficient amounts of metabolites to salvage the developing gametophytes. Nonetheless, our results show that both MIRO1 and MIRO2 affect mitochondrial function during female gametophyte development, and could also play a role in fertilization and early embryo development. Several knock-out studies of genes that encode mitochondria-targeted proteins show defects in gametogenesis. A particularly interesting mutant embryo sac phenotype observed in some of these mutants is the defect in fusion of polar nuclei (karyogamy) [35], [36], [37], [38], which we also observe in the *miro1* **^(+/−)^**/*miro2-2* **^(−/−)^** mutant. In a recent publication by Kägi and colleagues [39] it was demonstrated that a deleterious point mutation in mitochondria localized cysteinyl-tRNA synthetase (SYCO) and an ATP/ADP translocator AAC2 results in defects of polar nuclei fusion. Central cell mitochondria in *syco* and *aac2* plants lack cristae, indicating that SYCO and AAC2 is important for the structural integrity of the central cell mitochondria [39]. These results confirm that polar nuclei fusion in the central cell is a mitochondria dependant process. Investigations further showed that, the antipodal cells of the developing *syco* and *aac2* female gametophytes do not undergo PCD, suggesting that antipodal cell PCD is regulated by the adjacent central cell [39]. Our results are therefore in line with these findings where polar nuclei fusion is affected as a consequence of defects in mitochondrial function. It should also be investigated if the *miro1* **^(+/−)^**/*miro2-2* **^(−/−)^** embryo sacs contain antipodal cells that do not undergo PCD. The presence of EF-hands in the MIRO GTPases suggests a role for calcium ions in regulation of MIRO activity. Interestingly, during a large scale screen of mutants with impaired female gametophyte development, calmodulin binding proteins and Ca^2+^-binding proteins were reported and linked to defects in fusion of polar nuclei [40].

Research on MIRO orthologs in other model organisms (Drosophila, mammalian and human cell lines) has shown that MIRO GTPases facilitates mitochondrial movement and distribution along microtubuli in a Ca^2+^-dependent manner [41], [42]. It is therefore not unlikely that plant MIRO GTPases perform a similar role, despite the fact that mitochondria in plants mainly move along actin filaments. The observation that mitochondrial streaming in growing pollen tubes is disrupted in *miro1* **^(+/−)^** plants [14] supports this hypothesis. However, mitochondria in both *miro1* **^(+/−)^** pollen and embryos are enlarged, possibly due to increased fusion or the absence of fission events [14], [15]. It is therefore tempting to speculate that the observed defects in mitochondrial streaming may be a secondary effect due to inability of the transport machinery to shuttle enlarged mitochondria along actin strands. Furthermore, this suggests that plant MIRO GTPases play a significant role in mitochondrial fusion/fission events rather than movement. Saotome and colleagues showed that overexpression of human MIRO promoted the formation of elongated mitochondria seemingly by suppression of Dynamin-related protein1 (Drp1) mediated fission of mitochondria [43]. The Arabidopsis orthologs of human Drp1; DRP3A and DRP3B, have also been shown to regulate mitochondrial fission in a functionally redundant manner [44] and therefore a similar link between plant MIRO GTPases and plant DRPs may exists as well.

The fact that *MIRO1* and *MIRO2* are unequally redundant should be taken into consideration in future functional investigations. This especially applies to studying MIRO function during gamete development and function, since only the *miro1*/*miro2-2* haploid gametes display the full null phenotype. The supposed role for MIRO2 and thus possibly MIRO1 in plant stress signaling could also be the basis for future experiments. Finding MIRO protein partners will bring us closer to elucidating how MIRO GTPases regulate mitochondrial morphology and possibly mitochondrial distribution in plant cells.

## Supporting Information

File S1 Accession numbers for protein sequences used for phylogenetic analysis.
(PDF)

File S2 PCR genotyping analysis.
(PDF)
