# Supplementary material for: Arabidopsis thaliana MIRO1 and MIRO2 GTPases Are Unequally Redundant in Pollen Tube Growth and Fusion of Polar Nuclei during Female Gametogenesis
Source: PLoS One. 2011 Apr 11;6(4):e18530. doi: 10.1371/journal.pone.0018530 (PMC3073945; doi:10.1371/journal.pone.0018530)
Supplement: File S1 — Accession numbers for protein sequences used for phylogenetic analysis. (PDF) [file pone.0018530.s001.pdf]

Supplemental data S1: Accession numbers

Genes	Accession numbers	Organism
AtMIRO3	NP_187182.1	*Arabidopsis thaliana*
AlMIRO3	XP_002882407.1	*Arabidopsis lyrata subsp*
BrMIRO3	AC189329.1, genomic clone	*Brassica rapa* subsp pekinensis
AtMIRO1	NP_198106.1	*Arabidopsis thaliana*
BrMIRO1	AC189657.1, genomic clone	*Brassica rapa* subsp pekinensis
CpMIRO1	ABIM01011531.1, chromosome LG6 contig_11546	*Carica papaya*
PtMIRO1a	XP_002319545.1	*Populus trichocarpa*
PtMIRO1b	XP_002328439.1	*Populus trichocarpa*
RcMIRO1	XM_002512327.1 and AASG02000413.1 (genomic)	*Ricinus communis*
CmMIRO1	ABR67417.1	*Cucumis melo*
GmMIRO1	AK286579 (cDNA clone)	*Glycine max.*
FvMIRO1	CM001058.1, chromosome 6	*Fragaria vesca*
VvMIRO1	XP_002284757.1	*Vitis vinifera*
StMIRO1	BE344118, CV470227, BG600373, AK320284 (cDNA)	*Solanum lycopersicum and Solanum tuberosum.*
AtMIRO2	NP_567139.1	*Arabidopsis thaliana*
BrMIRO2	AC189426.1, genomic clone	*Brassica rapa* subsp pekinensis
CpMIRO2	ABIM01012020.1, chromosome LG6 contig_12035	*Carica papaya*
RcMIRO2	XP_002520752.1	*Ricinus communis*
PtMIRO2	XP_002306771.1	*Populus trichocarpa*
FvMIRO2	CM001054.1, chromosome 2	*Fragaria vesca*
GmMIRO2	ACUP01002127.1, chromosome 3 GLYMAchr_03_Cont2127	*Glycine max*
SlMIRO2	BABP01012491.1, DNA, contig: SlSBM_S02784_01	*Solanum lycopersicum*
VvMIRO2	XP_002275434.1	*Vitis vinifera*
OsMIRO1a	NP_001051665.1	*Oryza sativa (japonica cultivar-group)*
OsMIRO1b	AACV01002660.1, chromosome	*Oryza sativa (japonica cultivar-group)*

	1.	
TaMIRO1	AK332629.1 (cDNA clone)	*Triticum aestivum*
BdMIRO1a	ADDN01000017.1, chromosome 1	*Brachypodium distachyon* strain Bd21
BdMIRO1b	ADDN01000858.1, chromosome 3	*Brachypodium distachyon* strain Bd21
ZmMIRO1	ACG44216.1	*Zea mays*
ZmMIRO2	BT018890 (cDNA clone)	*Zea mays.*
PsMIRO1	DR498087, ES876866, ES248144 (cDNA)	*Picea sitchensis*
PpMIRO1	XP_001779282.1	*Physcomitrella patens subsp. patens*
PpMIRO2	XP_001778992.1	*Physcomitrella patens subsp. patens*
PpMIRO3	XP_001775852.1	*Physcomitrella patens subsp. patens*
PpMIRO4	XP_001767645.1	*Physcomitrella patens subsp. patens*
