# Supplementary material for: Arabidopsis thaliana MIRO1 and MIRO2 GTPases Are Unequally Redundant in Pollen Tube Growth and Fusion of Polar Nuclei during Female Gametogenesis
Source: PLoS One. 2011 Apr 11;6(4):e18530. doi: 10.1371/journal.pone.0018530 (PMC3073945; doi:10.1371/journal.pone.0018530)
Supplement: File S2 — PCR genotyping analysis. (PDF) [file pone.0018530.s002.pdf]

Supplemental data S2: PCR-genotyping of viable offspring on selection MS-media from self fertilized *miro1*$^{(+/-)}$/*miro2-2*$^{(+/-)}$ plants.

**Parental genotype**	**Total seedlings**	***miro1***$^{(+/-)}$/ ***miro2-2***$^{(+/-)}$	***miro1***$^{(+/-)}$/ ***miro2-2***$^{(-/-)}$	***miro1***$^{(+/-)}$/ ***miro2-2***$^{(-/-)}$ **(%)**	**Hypothesis**	χ^2	***P*(*P*<0.05)**
miro1$^{(+/-)}$/***miro2-2***$^{(+/-)}$ **x** ***miro1***$^{(+/-)}$/***miro2-2***$^{(+/-)}$	**80**	**63**	**17**	**21.3**	**1:2**	**5.262**	**0.0218**
